# Anti-hypertensive drugs deprescribing: an updated systematic review of clinical trials

**DOI:** 10.1186/s12875-021-01557-y

**Published:** 2021-10-20

**Authors:** Salvatore Crisafulli, Nicoletta Luxi, Raffaele Coppini, Annalisa Capuano, Cristina Scavone, Alessia Zinzi, Simona Vecchi, Graziano Onder, Janet Sultana, Gianluca Trifirò

**Affiliations:** 1 Department of Biomedical and Dental Sciences and Morphofunctional Imaging, University of Messina, Messina, Italy; 2 Department of Diagnostics and Public Health, University of Verona, P.le L.A. Scuro 10, 37134 Verona, Verona Italy; 3 Section of Pharmacology, Department of Neurology, Psychology, Drug Sciences and Child Health, University of Florence, Florence, Italy; 4 Department of Experimental Medicine, University of Campania “L. Vanvitelli”, Naples, Italy; 5 Campania Regional Centre for Pharmacovigilance and Pharmacoepidemiology, Naples, Italy; 6 Department of Epidemiology, Lazio Region- ASL Rome1, Rome, Italy; 7 Department of Cardiovascular and Endocrine-Metabolic Diseases and Aging, Istituto Superiore di Sanità, Rome, Italy; 8 Pharmacy Department, Mater Dei Hospital, Msida, Malta; 9 College of Medicine and Health, University of Exeter, Exeter, UK

**Keywords:** Deprescribing, Ant-hypertensive drugs, Multimorbidity, Polypharmacy

## Abstract

**Background:**

Polypharmacy is defined as the prescription of at least 5 different medicines for therapeutic or prophylactic effect and is a serious issue among elderly patients, who are frequently affected by multi-morbidity. Deprescribing is one of the proposed approaches to reduce the number of administered drugs, by eliminating those that are inappropriately prescribed. The aim of this systematic review is to provide an updated and systematic assessment of the benefit-risk profile of deprescribing of anti-hypertensive drugs, which are among the most commonly used drugs.

**Methods:**

MEDLINE, EMBASE and The Cochrane Library were searched for studies assessing the efficacy and safety of anti-hypertensive drugs deprescribing in the period between January, 12,016 and December, 312,019. The quality of randomized clinical trials (RCTs) was assessed using the GRADE approach for the evaluation of the main outcomes. The risk of bias assessment was carried out using the Cochrane risk-of-bias tool.

**Results:**

Overall, two RCTs were identified. Despite summarized evidence was in favor of anti-hypertensive deprescribing, the overall risk of bias was rated as high for each RCT included. According to the GRADE approach, the overall quality of the RCTs included was moderate regarding the following outcomes: systolic blood pressure < 150 mmHg after 12 weeks of follow-up, quality of life, frailty and cardiovascular risk.

**Conclusions:**

This updated systematic review of the efficacy and safety of anti-hypertensive treatment deprescribing found two recently published RCTs, in addition to the previous guideline of the National Institute for Health and Care Excellence (NICE). Evidence points towards non-inferiority of anti-hypertensive deprescribing as compared to treatment continuation, despite the quality of published studies is not high. High quality experimental studies are urgently needed to further assess the effect of deprescribing for this drug class in specific categories of patients.

**Supplementary Information:**

The online version contains supplementary material available at 10.1186/s12875-021-01557-y.

## Background

Polypharmacy is defined by the National Institute for Health as the administration of at least 5 different drugs for reducing the risk of morbidity and mortality [1]. It is a common issue among older persons, who are frequently affected by multi-morbidity that requires often complex pharmacological treatments [2]. In Italy, people aged ≥65 years received on average 7.7 different drugs in 2019, which increases to an average of 8.8 among persons aged ≥85 years [3]. One of the most serious issues associated with polypharmacy is the increased risk of drug-drug interactions (DDIs) that are clinically significant modifications in the pharmacokinetics and/or pharmacodynamics of one drug by the administration of another drug [4, 5]. DDIs can increase the risk of adverse drug reactions (ADRs), both in the primary or secondary care setting [6]. The chances of a DDI increase as the number of prescribed drugs increases [7–9]. As such, it is essential to implement preventive strategies to eliminate inappropriate drug prescribing, minimizing the overall amount of medicines taken on daily basis by the patients. Deprescribing is one potential approach to reduce inappropriate prescribing. Specifically, deprescribing is defined as the withdrawal or dose reduction of medications which are either inappropriate [10] or unnecessary [11], particularly among patients with polypharmacy. A systematic review and meta-analysis of studies evaluating the impact of deprescribing on mortality among the elderly found that among non-randomized studies, deprescribing significantly reduced mortality risk [odds ratio (OR) 0.32, 95% confidence interval (95% CI): 0.17–0.60)], while among randomized clinical trials (RCTs) this decreasing trend did not reach statistical significance (OR 0.82, 95% CI 0.61–1.11) [10, 12]. A more recent systematic review of RCTs suggested that the number of relevant trials was very low, with a high risk of bias and generally of low quality [13].

Of note, anti-hypertensive medications are of particular interest because they are very commonly used in Italy and can lead to ADRs especially among the elderly [3]. Age-related physiological changes, multimorbidity and polypharmacy may indeed alter pharmacokinetics and pharmacodynamics in elderly, thus altering significantly the effect of pharmacological treatment with advancing age [14]. Therefore, scaling down the number of medications taken, including anti-hypertensive drugs, by deprescribing, may lead to reduced adverse effects and improved quality of life in older people.

To date there is one guideline on clinical- and cost-effectiveness of stopping anti-hypertensive drugs [1]. This guideline identified very low quality evidence from 3 RCTs dated back to the ‘80s. Although such evidence suggested that for some people stopping anti-hypertensive drugs was significantly associated to the return to hypertension, the National Institute for Health and Care Excellence (NICE) Guideline Development Group concluded that deprescribing anti-hypertensive drugs may be beneficial for reducing polypharmacy and side effects, and for increasing quality of life, especially in “low risk” patients (e.g. people who have maintained blood pressure at normal levels for a long period of time or people with no history of cardiovascular events) [1]. Similarly, a Cochrane systematic review of six RCTs investigating the effect of anti-hypertensive medications in older people (i.e. aged ≥50 years) published in 2020 by Reeve et al. showed no evidence of an effect of discontinuing compared with continuing anti-hypertensives used for hypertension or primary prevention of cardiovascular disease on all-cause mortality and myocardial infarction. Furthermore, since the certainty of the evidence was low to very low, mainly because of the small size of the studies and low event rates, it was not possible to draw firm conclusions about the effect of anti-hypertensives deprescribing on these outcomes [12].

The aim of this systematic review was to synthesize current evidence on the assessment of the benefit-risk profile of anti-hypertensive drug deprescribing, including more recently published studies.

## Methods

### Search strategy

This systematic review was conducted in accordance with the Preferred Reporting Items for Systematic Reviews and Meta-Analyses (PRISMA) statement [15], but it was not registered on PROSPERO. The completed checklist can be found in Additional file 1. A search strategy was developed and applied to MEDLINE, EMBASE and The Cochrane Library to search for relevant studies (Additional file 2). The query search terms concerned anti-hypertensive drugs and discontinuation. Since the last systematic review on this topic searched studies until January, 42,016, our literature search was conducted from January, 12,016 to June, 302,020 [1]. The search results were exported into EndNote X9. Additional data was searched in clinical trial depositories (clinicaltrials.gov or EU Clinical Trials Register) or observational study registers (EU Post-Authorization Register).

The full inclusion/exclusion criteria for studies were developed systematically using the PICOS framework, i.e. population, intervention, comparator, outcome and study type, to answer the following research question: “what is the clinical effectiveness of stopping anti-hypertensive treatment?”. The set of inclusion/exclusion criteria is reported in Table 1. In short, both RCTs and cohort studies including people taking anti-hypertensive drugs as primary or secondary prevention for at least 1 year were eligible for inclusion. All studies must have been written in English, report clinical outcomes and must have compared drug deprescribing vs. continuation. Systematic reviews and meta-analyses were not included but were used as a source for snowball research. For studies that were excluded, the reason for exclusion was provided. After the elimination of duplicate studies, two authors independently read the title and abstract of all the identified studies, selecting which ones to consider for further evaluation. Two authors also read all full-texts independently for further assessment and final study inclusion. At all stages of the study, screening was carried out in parallel and any disagreement between the two authors was resolved by discussion until consensus was reached or by the intervention of a third author. In addition to this systematic search for articles, a snowball search was also conducted by identifying articles of interest through screening of the reference list of already identified studies.

**Table 1:** PICOS framework

Component	Description
Review question	What is the clinical- and cost-effectiveness of stopping antihypertensive treatment?
Area of scope	Effects of stopping treatment
Objective:	To evaluate the risks and benefits of stopping antihypertensive therapy to inform a recommendation
**P**opulation	People taking antihypertensive drugs as primary or secondary prevention for at least 1 year
**I**ntervention	Stopping anti-hypertensive agents (thiazides, beta blockers, alpha blockers, calcium-channel blockers, angiotensin-converting enzyme inhibitors, angiotensin receptor blockers)
**C**omparison	Continuing anti hypertension agents
**O**utcomes	**Critical:** • All-cause mortality • Cardiovascular mortality • Non-fatal myocardial infarction • Stroke • Quality of life • Hospitalization • Admission to care facility **Important:** • Blood pressure • Falls
**S**tudy design	RCTs and systematic reviews of RCTs
Exclusion	Pregnant women taking anti-hypertensives for secondary prevention Drugs used for other indications Duration of treatment less than 1 year

Abbreviations: *RCTs* randomized controlled trials

### Data extraction

Two authors independently collected information from selected studies concerning the following items: catchment area, study design, study population, exposure, outcomes and results. Where available, outcome estimates were reported as descriptive frequencies, odds ratios (OR), relative risk (RR) along with their 95% CI, and *p*-values.

### Risk of bias assessment

The quality of the identified studies was evaluated using validated scales. For RCTs, two authors independently assessed the risk of bias using the Cochrane risk-of-bias tool [16]. In case of disagreement, the final judgment was made after reaching consensus by involving a third review author. This tool consists of 6 items; for each of them, a quality rating was carried out and reported as a “low”, “high” or “unclear” risk of bias, based on the presence of sufficient information from the published study.

We used the Grading of Recommendations, Assessment, Development and Evaluation (GRADE) methodology to assess and rate the quality of body of evidence for each outcome on the basis of study design, inconsistency, indirectness and imprecision [17]. The certainty of evidence in the effect estimates for the body of evidence was assessed as high, moderate, low, or very low. We created summary of findings tables using GRADE’s electronic tool GRADEpro GDT (www.gradepro.org).

## Results

### Study selection

**Fig. 1:**
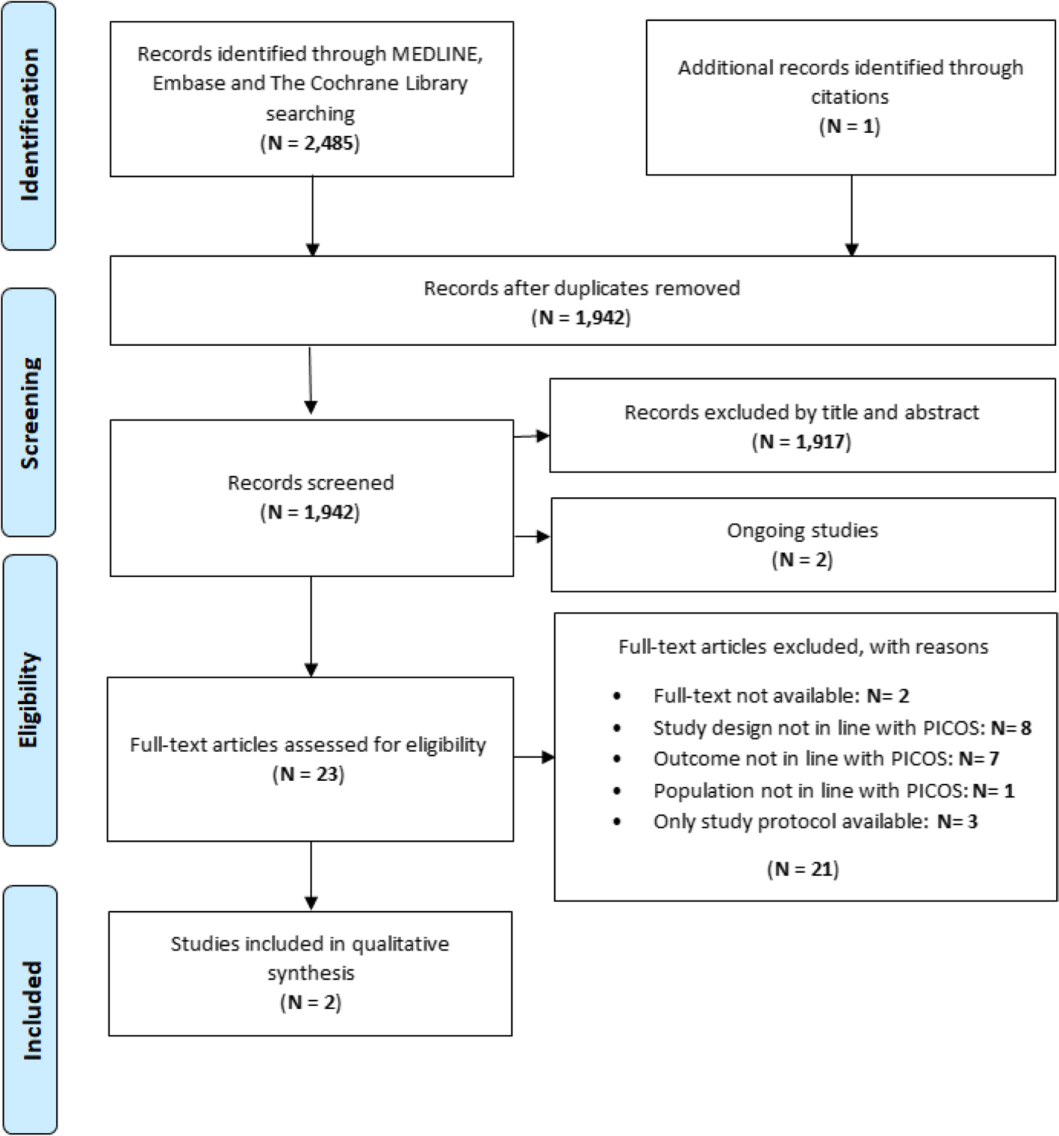
PRISMA flowchart showing the process of literature search and study selection

The flow-chart outlining study selection of each drug class is shown in Fig. 1. The initial literature search yielded a total of 2486 studies. After removal of duplicates (*N* = 544), 1942 abstracts were screened and of these only 23 full-text articles were selected for further evaluation. Of the 23 selected studies, the full-text was not available in 2 studies, 18 were not in line with the PICO and for 1 only the protocol was available; only 2 RCTs were of interest for our investigation and no observational studies meeting our inclusion criteria were found. Reasons for exclusion are described in detail in Additional file 3.

### Characteristics of studies on anti-hypertensive drug deprescribing

Table 2 presents the characteristics of the studies included in the NICE guideline and of the studies included in this systematic review, summarizing the findings for all relevant outcomes evaluated in the RCTs. Overall, evidence from the three RCTs included in the NICE guideline suggested a clinical benefit associated with stopping treatment, as compared to continuing anti-hypertensive treatment, in terms of cardiovascular mortality. Moreover, evidence indicated that treatment withdrawal was associated with a clinical harm for return to hypertension (i.e. a rise in blood pressure to above the threshold for diagnosing hypertension) and a clinical benefit for maintaining target blood pressure [18–20]. However, a significant proportion of people randomized to treatment discontinuation did not return to hypertension, thus suggesting that a considerable proportion of patients treated with anti-hypertensive drugs may stop therapy without returning to hypertension. The overall quality of the evidence was very low, mainly due to the small sample size and to the low event rates [1].

**Table 2:** Characteristics of the studies included in the NICE guideline and of the studies included in the current study

Study, Catchment area	Study design	Study population	Exposure	Outcomes	Results
**NICE Guidelines**					
Freis et al., 1975 [18] United States	Double-blind randomized clinical trial	Adult veterans hospitalized prior to treatment for hypertension, with average diastolic blood pressure < 95 mmHg for 2 or more years and treated with anti-hypertensives for primary prevention for ≥2 years	Discontinuation of anti-hypertensive drugs and allocation to placebo (*N* = 60) vs. Continuation of anti-hypertensive drugs (*N* = 26)	- Cardiovascular mortality after 18 months of follow-up; - Non-fatal congestive heart failure after 18 months of follow-up; - Atrial fibrillation after 18 months of follow-up; - Right bundle block after 18 months of follow-up	During the 18 months of follow-up, 51 people (85%) in the intervention group were removed from the trial: 42 (70%) because of return to increased arterial pressures and 6 (10%) because of major cardiovascular complications. Nine (15%) of the placebo patients remained normotensive.
Greenberg et al., 1986 [19] England	Open label randomized clinical trial	Adult people with mild hypertension (diastolic blood pressure 90–109 mmHg) and treated with anti-hypertensive drugs for primary prevention for 5.5 years	Discontinuation of anti-hypertensives (bendrofluazide or propanololol) (*N* = 783) vs. continuation of anti-hypertensives (bendrofluazide or propanololol) (*N* = 837)	Maintaining target blood pressure (diastolic blood pressure < 90 mmHg) after 2 years of follow-up	396 people (24.4%) completed the 2 year follow up. Mean blood pressures rose- rapidly after the withdrawal of active treatment, and between nine months and one year after stopping treatment the antihypertensive effect almost disappeared. After stopping propranolol pressure tended to increase less quickly in older people than in younger.
Maland et al., 1983 [20] United States	Double-blind randomized clinical trial	Adult living in the community, with mild hypertension (diastolic blood pressure average 90 mmHg or less for 1 year) and treated with anti-hypertensives for primary prevention for ≥1 year	Discontinuation of anti-hypertensives (chlorthalidone, hydrothiazide, triamterene) and allocated to placebo (*N* = 31) vs. Continuation of anti-hypertensives (chlorthalidone, hydrothiazide, triamterene) (*N* = 31)	- Cardiovascular mortality after 1 year of follow-up - Non-fatal myocardial infarction after 1 year of follow-up - Transient ischaemic attack after 1 year of follow-up - Return to hypertension (diastolic blood pressure > 95 mmHg) after 1 year of follow-up	59 people (95.2%) completed the 1 year follow up. Nine patients reverted to elevated blood pressure: eight from the placebo group and one from the active treatment group (*p* = 0.03). The average time to reversion was 27 weeks, with a range of 15 to 48 weeks. Elevated pressures in all reverters returned to normotensive levels within 1 month of restarting the pre-trial medication at the dose-level previously used
**Current study**					
Sheppard et al., 2020 [21] United Kingdom	Open label randomized clinical trial	Patients aged ≥80 years with blood pressure < 150 mmHg and who had received ≥2 anti-hypertensives for ≥1 year. Enrolled patients: 569	Medication reduction (*N* = 282 patients) vs. Standard clinical practice (*N* = 287 patients)	- Maintaining systolic blood pressure < 150 mmHg after 12 weeks of follow-up; - % of patients in the intervention group who maintained the reduced therapeutic regimen; - Differences between the two groups in frailty, quality of life, adverse events and changes in blood pressure over 12 weeks.	- 12-week follow-up: 229 (86,4%) patients in the intervention group and 236 (87,7%) patients with standard therapy had blood pressure < 150 mmHg (RR = 0.98 [97.5% 1-sided CI, 0.92- ∞]); - Medication reduction was maintained in 187 (66.3%) patients in the intervention group; - At week 12, mean blood pressure was 133.7 (95% CI, 131.7–135.6) mmHg in the intervention group and 130.8 (95% CI, 128.9–132.7) mmHg in the control group; - No significant differences were found between the two groups in terms of frailty, quality of life and adverse events.
Luymes et al., 2018 [22] The Netherlands	Open label randomized clinical trial	Patients aged from 40 to 70 years, without established cardiovascular diseases and who had been using anti-hypertensive or lipid-lowering drugs for at least 1 year	Medication reduction (*N* = 492 patients) vs. Usual care (*N* = 575 patients)	- Increase in participants’ predicted 10-year cardiovascular risk after 2 years of follow-up; - Systolic and diastolic blood pressure differences between the two groups	A 2-year increase in cardiovascular risk was observed in both groups (from 4.7 to 6.7% for the intervention group and from 5.1 to 7.0% for the usual care group). Since only a difference of 0.1 (95% CI: − 0.3 to 0.6) percentage points was detected, non-inferiority was established. Moreover, systolic and diastolic blood pressure was higher in the intervention group than in the usual care group (*p*-value < 0.01 for both comparisons).

Abbreviations: *CI* confidence interval; *NICE* National Institute for Health and Care Excellence; *OR* odds ratio; *RR* relative risk

Both the two RCTs included in this systematic review were open label RCTs [21, 22].

The RCT conducted in England [21] recruited 569 patients aged ≥80 years, with a blood pressure < 150 mmHg and who had received at least 2 anti-hypertensive drugs for at least 1 year. Of these patients, 282 had a medication reduction, while 287 patients underwent standard clinical practice. The main outcome was systolic blood pressure < 150 mmHg after 12 weeks of follow-up, while secondary outcomes included the proportion of patients in the intervention group who maintained medication reduction and differences between the two groups in frailty, quality of life, adverse event and changes in blood pressure over 12 weeks. Overall, at 12 weeks of follow-up, 229 (86.4%) patients in the intervention group and 236 (87.7%) patients with standard therapy group had blood pressure < 150 mmHg with a RR of 0.98 (97.5% CI: 0.92-∞). Anti-hypertensive reduction was maintained in 187 (66.3%) patients in the intervention group. Mean blood pressure was 133.7 (95% CI: 131.7–135.6) mmHg in the intervention group and 130.8 (95% CI: 128.9–132.7) mmHg in the control group. No statistically significant differences between the two groups in terms of frailty, quality of life and adverse events were observed.

Similarly to the 3 RCTs included in the NICE guidelines, the risk of bias was rated as high for both the RCTs included in this systematic review, mainly due to the lack of blinding of participants and personnel and the lack of blinding of outcome assessment (Table 3). The certainty of evidence was moderate for all outcomes (Table 4).

**Table 3:** Risk of bias assessment for the studies included in the NICE guideline and for the studies included in the current study

Study	Random sequence generation	Allocation concealment	Blinding participants and personnel	Blinding of outcome assessment	Incomplete outcome data	Selective data reporting
**NICE Guidelines**						
Freis, 1975 [18]	**?**	**–**	**+**	**?**	**+**	**–**
Greenberg, 1986 [19]	**+**	**?**	**?**	**?**	**–**	**?**
Maland, 1983 [20]	**?**	**?**	**+**	**?**	**+**	**–**
**Current study**						
Sheppard, 2020 [21]	**+**	**+**	**–**	**–**	**+**	**+**
Luymes, 2018 [22]	**+**	**+**	**–**	**–**	**+**	**+**

Abbreviations: *NICE* National Institute for Health and Care Excellence

Legend: Green symbol = low risk of bias; yellow symbol = unclear risk of bias; red symbol = high risk of bias

**Table 4:** Summary of findings for the main outcomes using the GRADE methodology

Certainty assessment							Number of patients		Effect		Certainty
№ of studies	Study design	Risk of bias	Inconsistency	Indirectness	Imprecision	Other considerations	Anti-hypertensives withdrawal	Anti-hypertensives continuation	Relative (95% CI)	Absolute (95% CI)	
Number of patients with systolic blood pressure < 150 mmHg (mean follow up: 12 weeks)											
1 [21]	Open label RCT	Serious	Not serious	Not serious	Not serious	None	229/265 (86.4%)	236/269 (87.7%)	**RR 0.98** (0.92 to 1.05)	**18 fewer per 1000** (70 fewer to 44 more)	⨁⨁⨁◯ MODERATE
Quality of life (mean follow up: 12 weeks; assessed with: EQ-5D-5L Index)											
1 [21]	Open label RCT	Serious	Not serious	Not serious	Not serious	None	260	263	–	MD 0.01 lower (0.03 lower to 0.01 higher)	⨁⨁⨁◯ MODERATE
Frailty (assessed with: Frailty Index)											
1 [21]	Open label RCT	Serious	Not serious	Not serious	Not serious	None	282	287	–	MD 0.00003 lower (0.0005 lower to 0.005 higher)	⨁⨁⨁◯ MODERATE
Cardiovascular risk											
1 [22]	Open label RCT	Serious	Not serious	Not serious	Not serious	None	492	575	–	MD 0.1 higher (0.4 lower to 0.7 lower)	⨁⨁⨁◯ MODERATE

Abbreviations: *CI* Confidence interval; *RCT* randomized controlled trial; *RR* Risk ratio; *MD* Mean difference

The findings of this study suggest that reducing anti-hypertensive drugs in elderly hypertensive patients is safe and not associated with significant change in blood pressure control.

Another RCT, conducted in the Netherlands by Luymes et al. [22], enrolled 1067 patients aged between 40 and 70 years, with no history of cardiovascular diseases and who used anti-hypertensive or lipid-lowering drugs for at least 1 year. Participants were randomized either to receive an intervention of medication reduction or to continue with usual care. The primary outcome was the increase in 10-year cardiovascular risk in the 2 years of follow-up, while systolic and diastolic blood pressure was included among secondary outcomes. Overall, 2-year increase in cardiovascular risk was observed in both groups (from 4.7 to 6.7% for the intervention group and from 5.1 to 7.0% for the usual care group). Since only a difference of 0.1 (95% CI: − 0.3 to 0.6) percentage points was detected, non-inferiority was established. Moreover, systolic and diastolic blood pressure was higher in the intervention group than in the usual care group (*p*-value < 0.01 for both comparisons). The certainty of evidence was moderate for cardiovascular outcomes (Table 4).

This study indicated that an attempt to deprescribe anti-hypertensive medications in patients with low cardiovascular risk is safe in the short term, while blood pressure has to be monitored after anti-hypertensive drug withdrawal.

### Summary of implications for deprescribing

Overall, the RCTs concerning anti-hypertensive drug deprescribing, arguably the most robust study design to answer the research questions, were in favor of deprescribing; these studies were of moderate quality [21, 22]. However, further higher quality scientific evidence is needed.

## Discussion

This systematic review has summarized data from two RCTs including 1636 adults treated with anti-hypertensive drugs. We did not find observational studies meeting the inclusion criteria for this systematic review. One of the main findings of this systematic review was the dearth of robust information on the risks and benefits of deprescribing of anti-hypertensive drugs. Indeed, the review carried out only identified two new studies. Furthermore, the quality of the included studies was generally not high. Robust studies on deprescribing are needed to update and inform clinical guidelines.

Evidence from the included studies indicates that, in terms of blood pressure control, a strategy of medication reduction is non-inferior compared to standard care [21, 22]. However, these trials focused on two different specific populations and evaluated different outcomes, thus making it difficult to draw conclusions generalizable to individuals not included in such populations. Specifically, the OPTIMISE trial enrolled patient aged at least 80 years who were prescribed with two or more anti-hypertensive drugs for at least 12 months, selected based on the general practitioner opinion that they might benefit from deprescribing. Although the patient population in this study was generalizable to primary care, this trial did not establish whether or not anti-hypertensive drug deprescribing should be attempted or which patients should be targeted with such an intervention [21]. On the other hand, the ECSTATIC study enrolled younger patients, aged between 40 and 70 years, without history of cardiovascular diseases and with low risk of future cardiovascular diseases. In this pragmatic trial, the increase in predicted 10-year cardiovascular risk in the 2 years after the first visit was assessed, with the main objective to answer the question of whether a structured deprescribing strategy in low-cardiovascular-risk patients is effective when implemented in general practice. Moreover, in this study medication reduction was part of a medication review but not specifically mandated; the choice to leave the decision to deprescribe to the patient and their general practitioners and the choice to use an active control group may have resulted in an underestimation of the effect of the intervention on cardiovascular risk [22].

Overall, findings from the present systematic review are in line with the NICE guideline on anti-hypertensive drugs deprescribing, suggesting benefits, in terms of blood pressure management, especially low-risk patients (i.e. patients without history of cardiovascular events and whose blood pressure was maintained at normal levels for a long period of time). NICE guidelines on managing multi-morbidity in terms of anti-hypertensive prescribing state that there was a reduced risk of cardiovascular outcomes on discontinuing anti-hypertensive treatment, compared to continuing it [1]. On discontinuation, there was a risk of reverting to high blood pressure, but this was found to be uncommon. However, these guidelines focus on simply discontinuing a drug rather than the broader range of deprescribing options, such as dose reduction and other changes to the dosing regimen. In addition, these guidelines do not clearly state which clinical characteristics would make a person eligible for discontinuation. The studies upon which the guidelines were based were considered to be of low to moderate quality and date back to ‘80s.

In this regard, it is noteworthy that only two RCTs meeting our inclusion criteria have been published on this topic since 1986 [23], i.e. the year of publication of the last study included in the NICE guideline, highlighting the lack of evidence on such an important topic for such a long period of time.

Findings from this systematic review are also in line with those of the Cochrane systematic review published by Reeve et al. in June 2020 [12]. In this paper, the authors used different inclusion criteria, including also studies in which patients were treated with anti-hypertensive drugs for less than 1 year, excluding RCTs in which anti-hypertensive drugs were used for secondary prevention and setting age limits (i.e. only studies concerning people aged ≥50 years were included). They searched six bibliographic databases until April 2019 and included six RCTs in their systematic review: five of them were published between 1977 and 1997 and one in 2015. Only one [20] of these RCTs met the criteria established by our PICO and was already included in the NICE guidelines. Overall, evidence from this systematic review demonstrated that anti-hypertensive drug discontinuation had no effects on all-cause mortality, myocardial infarction or stroke, although there was low or very low certainty in these results [12]. As the authors stated, one of the main limitations of their systematic review was that five out of the six included RCTs were published more than 20 years ago. Indeed, over the past 20 years, several changes in standards of treatment and population risk factors have occurred and the number of the oldest old as well as the prevalence of polypharmacy among older adults has increased, thus affecting the applicability of the evidence to the current population of older adults.

Further information is needed on which patients can benefit most from deprescribing anti-hypertensive drugs because these drugs are widely used long-terms and over-used, especially among older persons [24]. Although these drugs are generally considered to be well-tolerated, they are still associated with ADRs, the risk of which may be increased also due to DDIs. For example, anti-hypertensives are associated with a higher risk of falls among elderly persons that are treated with several anti-hypertensives [25] as well as with other drugs with hypotensive effects such as trazodone [26] and tricyclic antidepressants [27].

An observational study conducted among elderly hospitalized patients found that 15% of moderately severe interactions occurred due to the use of angiotensin converting enzyme inhibitors and loop diuretics; the number of DDIs was proportionally associated with the risk of death [28]. A more recent observational study found that cardiovascular drugs are by far the most frequently involved drug class in DDI occurrence also in primary care [29]. If successfully implemented, deprescribing as a strategy to reduce polypharmacy may also lead to risk reduction of ADRs and DDIs and ultimately to optimization of healthcare costs [].

The present study has several strengths. The findings of this study are based on a systematic and independent review of available literature. This study has also built on previous systematic reviews to update them and avoid duplicating work. However, some study limitations warrant cautions. Although only high quality RCTs can provide robust evidence on the safety and efficacy of deprescribing, only two RCTs of medium quality were found. The evidence on deprescribing from observational studies is generally weak as drug discontinuation/switch to a lower dose may occur for several reasons which may be difficult to identify (e.g. short life expectancy with focus on palliative care, unwillingness to continue therapy, onset of ADRs, etc.) and may act as confounders that cannot be fully adjusted in naturalistic setting. Furthermore, due to the high risk of bias and the heterogeneity of the study designs and outcomes of the included studies, it was not possible to conduct a meta-analysis [31].

Future studies should address the impact of deprescribing of commonly used drugs, such as anti-hypertensive medications, in specific subpopulations (e.g. elderly patients) using clear definitions of deprescribing and clearly reporting which patients were eligible for deprescribing. Such studies should consider clinically meaningful outcomes when evaluating the risks and benefits of discontinuations, such as the risk of outcomes linked to mortality (e.g. hypotension, falls, pneumonia etc.) as well as focusing on populations at highest risk of ADRs, such as persons with well-defined polypharmacy and multi-morbidity, as well as elderly persons. Such studies should ideally be RCTs, as these studies are best-suited to measure the effectiveness of interventions, including deprescribing interventions. While observational studies may be useful to describe the real-world use of anti-hypertensives, the discontinuation of these drugs in a routine clinical setting may be driven by unmeasured confounders, such as disease severity and prescriber willingness to attempt deprescribing that cannot be totally addressed. The ideal study design to evaluate the impact of deprescribing may be a pragmatic study.

## Conclusions

This updated systematic review of the risks and benefits of anti-hypertensive drugs deprescribing found that there are only two RCTs beyond those included in the guideline published in 2016 by NICE. Although included studies are of moderate quality, the overall evidence seems to suggest a positive effect of anti-hypertensive drugs deprescribing, in line with the NICE guideline. Given how widely used and potentially over-used anti-hypertensives are, especially among the elderly, high quality experimental studies are urgently needed to measure the effect of deprescribing interventions, as they can reduce the risk of DDIs, thereby improving the safety of drug use.

## Additional files

**Additional file 1.** .pdf: Preferred Reporting Items for Systematic Reviews and Meta-Analyses (PRISMA) checklist.

**Additional file 2.** .pdf: Search strategy.

**Additional file 3.** .pdf: Excluded studies, with reasons for exclusion.

## Data Availability

All data generated and analysed during this study are included in this manuscript and the supporting file.

## Abbreviations

ADRs: Adverse drug reactions
DDIs: Drug-drug interactions
GRADE: Grading of recommendations, assessments, development and evaluation
MD: Mean difference
NICE: National institute for health and care excellence
OR: Odds ratio
PRISMA: Preferred reporting items for systematic reviews and meta-analyses
RCTs: Randomized clinical trials
RR: Relative risk
95% CI: 95% Confidence interval
