# Supplementary material for: Anti-hypertensive drugs deprescribing: an updated systematic review of clinical trials
Source: BMC Fam Pract. 2021 Oct 20;22:208. doi: 10.1186/s12875-021-01557-y (PMC8527765; doi:10.1186/s12875-021-01557-y)
Supplement: Supplementary file 2 — Additional file 2. .pdf: Search strategy. [file 12875_2021_1557_MOESM2_ESM.pdf]

Additional file 2. Search strategy

The Cochrane Library

#1 MeSH descriptor: [Hypertension] this term only
#2 hypertens*:ti,ab
#3 ((elevat* or high or increas*) near/3 blood adj pressur*):ti,ab
#4 #1 OR #2 OR #3
#5 (thiazide* or bendrofluazide or bendroflumethazide or aprinox or neo-naclex or chlorthalidone or chlortalidone or hygroton or cyclopenthiazide or navidrex or indapamide or natrilix or metolazone or xipamide or diurexan or hydrochlorthiazide or hydrochlorothiazide or neo-naclex-k):ti,ab,kw
#6 (calcium near/3 (block* or inhibit* or antagonist*)):ti,ab,kw
#7 (diltiazem or optil or tildiem or adizem or angitil or calcicard or dilcardia or dilzem or slozem or viazem or zemtard or verapamil or zolvera or cordilox or securon or univer or verapress or vertab):ti,ab,kw
#8 (amlodipine or amlostin or istin or exforge or felodipine or plendil or lacidipine or motens or lercanidipine or zanidip or nicardipine or cardene or nifedipine or adalat or nimodipine or nimotop or coracten or adipine or fortipine or tensipine or valni or nifedipress):ti,ab,kw
#9 (propranolol or angilol or inderal-la or half-inderal or inderal or bedranol or syprol or prograne or slo-pro or acebutolol or sectral or atenolol or tenormin or bisoprolol or cardicor or emcor or carvedilol or eucardic or celiprolol or celectol or co-tenidone or tenoret or tenoretic or labetalol or trandate or metoprolol or betaloc or lopresor or nadolol or corgard or nebivolol or nebilet or hypoloc or oxprenolol or trasicor or slow-trasicor or pindolol or visken or viskaldix or timolol or betim):ti,ab,kw
#10 ((beta or b) near/3 (block* or antagonist*)):ti,ab,kw
#11 ((angiotensin near3 (receptor* near/2 (antagonist* or blocker*))) or arb or arbs):ti,ab,kw
#12 (candesartan or amias or eprosartan or teveten or irbesartan or aprovel or coaprovel or losartan or cozaar or cozaar-comp or olmesartan or olmetec or sevikar or

telmisartan or micardis or valsartan or diovan or co-diovan or azilsartan or edarbi):ti,ab,kw
#13 ((ace or acei or ((angiotensin adj converting anear/2 enzyme*) or ace or kininase)) near/2 (inhibit* or antagonist*)):ti,ab
#14 (captopril or ecopace or kaplon or capoten or co-zidocapt or capto-co or capozide or cilazapril or vascace or enalapril or ednyt or innovace or innozide or fosinopril or imidapril or tanatril or lisinopril or zestril or carace or zestoretic or moexipril or perdix or perindopril or coversyl or quinapril or quinil or accupro or accuretic or ramipril or tritace or triapin or trandolapril or gopten or tarka):ti,ab
#15 MeSH descriptor: [Antihypertensive Agents] this term only
#16 (antihypertens* near/2 (drug* or agent* or treat* or therap* or intervention*)):ti,ab,kw
#17 {OR #5-#16}
#18 MeSH descriptor: [Polypharmacy] this term only
#19 polypharmacy:ti,ab
#20 MeSH descriptor: [Patient Compliance] this term only
#21 MeSH descriptor: [Medication Adherence] this term only

#22 (deprescri* or unprescri* or cease* or ceasing* or cessation* or withdraw* or discontinu* or stop* or intermittent or demand):ti,ab
#23 {OR #18-#22}
#24 #4 AND #17 AND #23 with Cochrane Library publication date from Jan 2016 to present, in Trials

OVID MEDLINE

#1 exp hypertension/
#2 hypertens*.ti,ab.
#3 ((elevat* or high or increas*) adj3 blood adj pressur*).ti,ab.
#4 #1 or #2 or #3
#5 exp *thiazides/
#6 (thiazide* or bendrofluazide or bendroflumethazide or aprinox or neo-naclex or chlorthalidone or chlortalidone or hygroton or cyclopenthiazide or navidrex or indapamide or natrilix or metolazone or xipamide or diurexan or hydrochlorthiazide or hydrochlorothiazide or neo-naclex-k).ti,ab.
#7 exp *calcium channel blockers/
#8 (calcium adj3 (block* or inhibit* or antagonist*)).ti,ab.
#9 (diltiazem or optil or tildiem or adizem or angitil or calcicard or dilcardia or dilzem or slozem or viazem or zemtard or verapamil or zolvera or cordilox or securon or univer or verapress or vertab).ti,ab.
#10 (amlodipine or amlostin or istin or exforge or felodipine or plendil or lacidipine or motens or lercanidipine or zanidip or nicardipine or cardene or nifedipine or adalat or nimodipine or nimotop or coracten or adipine or fortipine or tensipine or valni or nifedipress).ti,ab.
#11 exp *adrenergic beta-antagonists/
#12 (propranolol or angilol or inderal-la or half-inderal or inderal or bedranol or syprol or prograne or slo-pro or acebutolol or sectral or atenolol or tenormin or bisoprolol or cardicor or emcor or carvedilol or eucardic or celiprolol or celectol or co-tenidone or tenoret or tenoretic or labetalol or trandate or metoprolol or betaloc or lopresor or nadolol or corgard or nebivolol or nebilet or hypoloc or oxprenolol or trasicor or slow-trasicor or pindolol or visken or viskaldix or timolol or betim).ti,ab.
#13 ((beta or b) adj3 (block* or antagonist*)).ti,ab.
#14 exp *angiotensin ii type 1 receptor blockers/ or *angiotensin ii type 2 receptor blockers/
#15 ((angiotensin adj3 (receptor* adj2 (antagonist* or blocker*))) or arb or arbs).ti,ab.
#16 (candesartan or amias or eprosartan or teveten or irbesartan or aprovel or coaprovel or losartan or cozaar or cozaar-comp or olmesartan or olmetec or sevikar or telmisartan or micardis or valsartan or diovan or co-diovan or azilsartan or edarbi).ti,ab.
#17 exp *angiotensin-converting enzyme inhibitors/
#18 ((ace or acei or ((angiotensin adj converting adj2 enzyme*) or ace or kininase)) adj2 (inhibit* or antagonist*)).ti,ab.
#19 (captopril or ecopace or kaplon or capoten or co-zidocapt or capto-co or capozide or cilazapril or vascace or enalapril or ednyt or innovace or innozide or fosinopril or imidapril or tanatril or lisinopril or zestril or carace or zestoretic or moexipril or perdix or perindopril or coversyl or quinapril or quinil or accupro or accuretic

or ramipril or tritace or triapin or trandolapril or gopten or tarka).ti,ab.
#20 *antihypertensive agents/
#21 (antihypertens* adj2 (drug* or agent* or treat* or therap* or intervention*)).ti,ab.
#22 or/#5-#21
#23 #4 and #22
#24 (deprescri* or de-prescri*).ti,ab.
#25 (stop adj3 (criteria or criterion or rule or standard or benchmark or bench mark or decision* or take or taking)).ti,ab.
#26 (discontinu* or withdraw* or cessat* or down-titrat* or step-down or "step down").ti.
#27 ((discontinu* or withdraw* or cessat* or down-titrat* or step-down or "step down" or stop* or cease* or taper*) adj2 (dose* or drug* or treatment* or therap* or medicat* or intervention*)).ti,ab.
#28 polypharmacy/
#29 polypharmacy.ti,ab.
#30 *medication adherence/
#31 *patient compliance/
#32 *treatment refusal/
#33 (adheren* or nonadheren* or non-adheren* or non adheren* or complian* or noncomplian* or non-complian* or non complian*).ti.
#34 ((adheren* or nonadheren* or non-adheren* or non adheren* or complian* or noncomplian* or non-complian* or non complian* or persist*) adj2 (patient* or participant* or dose* or drug* or treatment* or therap* or medicat* or intervention*)).ti,ab.
#35 or/#24-#34
#36 #23 and #35
#37 letter/
#38 editorial/
#39 news/
#40 exp historical article/
#41 anecdotes as topic/
#42 comment/
#43 case report/
#44 (letter or comment*).ti.
#45 #37 or #38 or #39 or #40 or #41 or #42 or #43 or #44
#46 randomized controlled trial/ or random*.ti,ab.
#47 #45 not #46
#48 animals/ not humans/
#49 exp animals, laboratory/
#50 exp animal experimentation/
#51 exp models, animal/

#52 exp rodentia/
#53 (rat or rats or mouse or mice).ti.
#54 #47 or #48 or #49 or #50 or #51 or #52 or #53
#55 #36 not #54
#56 limit 55 to yr="2016 -Current"

EMBASE

#1 exp *hypertension/
#2 hypertens*.ti,ab.
#3 ((elevat* or high or increas*) adj3 blood adj pressur*).ti,ab.
#4 1 or 2 or 3
#5 exp *thiazide diuretic agent/
#6 (thiazide* or bendrofluazide or bendroflumethazide or aprinox or neo-naclex or chlorthalidone or chlortalidone or hygroton or cyclopenthiazide or navidrex or indapamide or natrilix or metolazone or xipamide or diurexan or hydrochlorthiazide or hydrochlorothiazide or neo-naclex-k).ti,ab.
#7 (calcium adj3 (block* or inhibit* or antagonist*)).ti,ab.
#8 (diltiazem or optil or tildiem or adizem or angitil or calcicard or dilcardia or dilzem or slozem or viazem or zemtard or verapamil or zolvera or cordilox or securon or univer or verapress or vertab).ti,ab.
#9 (amlodipine or amlostin or istin or exforge or felodipine or plendil or lacidipine or motens or lercanidipine or zanidip or nicardipine or cardene or nifedipine or adalat or nimodipine or nimotop or coracten or adipine or fortipine or tensipine or valni or nifedipress).ti,ab.
#10 exp *beta adrenergic receptor blocking agent/
#11 (propranolol or angilol or inderal-la or half-inderal or inderal or bedranol or syprol or prograne or slo-pro or acebutolol or sectral or atenolol or tenormin or bisoprolol or cardicor or emcor or carvedilol or eucardic or celiprolol or celectol or co-tenidone or tenoret or tenoretic or labetalol or trandate or metoprolol or betaloc or lopresor or nadolol or corgard or nebivolol or nebilet or hypoloc or oxprenolol or trasicor or slow-trasicor or pindolol or visken or viskaldix or timolol or betim).ti,ab.
#12 ((beta or b) adj3 (block* or antagonist*)).ti,ab.
#13 exp *angiotensin receptor antagonist/
#14 ((angiotensin adj3 (receptor* adj2 (antagonist* or blocker*))) or arb or arbs).ti,ab.
#15 (candesartan or amias or eprosartan or teveten or irbesartan or aprovel or coaprovel or losartan or cozaar or cozaar-comp or olmesartan or olmetec or sevikar or telmisartan or micardis or valsartan or diovan or co-diovan or azilsartan or edarbi).ti,ab.
#16 exp *dipeptidyl carboxypeptidase inhibitor/
#17 ((ace or acei or ((angiotensin adj converting adj2 enzyme*) or ace or kininase)) adj2 (inhibit* or antagonist*)).ti,ab.
#18 (captopril or ecopace or kaplon or capoten or co-zidocapt or capto-co or capozide or cilazapril or vascace or enalapril or ednyt or innovace or innozide or fosinopril or imidapril or tanatril or lisinopril or zestril or carace or zestoretic or moexipril or perdix or perindopril or coversyl or quinapril or quinil or accupro or accuretic or ramipril or tritace or triapin or trandolapril or gopten or tarka).ti,ab.

#19	*antihypertensive agent/
#20	(antihypertens* adj2 (drug* or agent* or treat* or therap* or intervention*)).ti,ab.
#21	exp *calcium channel blocking agent/
#22	#5 or #6 or #7 or #8 or #9 or #10 or #11 or #12 or #13 or #14 or #15 or #16 or #17 or #18 or #19 or #20 or #21
#23	#4 and #22
#24	(deprescri* or de-prescri*).ti,ab.
#25	(stop adj3 (criteria or criterion or rule or standard or benchmark or bench mark or decision* or take or taking)).ti,ab.
#26	(discontinu* or withdraw* or cessat* or down-titrat* or step-down or "step down").ti.
#27	((discontinu* or withdraw* or cessat* or down-titrat* or step-down or "step down" or stop* or cease* or taper*) adj2 (dose* or drug* or treatment* or therap* or medicat* or intervention*)).ti,ab.
#28	*polypharmacy/
#29	polypharmacy.ti,ab.
#30	*patient compliance/ or *medication compliance/
#31	*treatment refusal/
#32	(adheren* or nonadheren* or non-adheren* or non adheren* or complian* or noncomplian* or non-complian* or non complian*).ti.
#33	((adheren* or nonadheren* or non-adheren* or non adheren* or complian* or noncomplian* or non-complian* or non complian* or persist*) adj2 (patient* or participant* or dose* or drug* or treatment* or therap* or medicat* or intervention*)).ti,ab.
#34	or/#24-#33
#35	#23 and #34
#36	letter.pt. or letter/
#37	note.pt.
#38	editorial.pt.
#39	case report/ or case study/
#40	(letter or comment*).ti.
#41	#36 or #37 or #38 or #39 or #40
#42	randomized controlled trial/ or random*.ti,ab.
#43	#41 not #42
#44	animal/ not human/
#45	nonhuman/
#46	exp animal experiment/
#47	exp experimental animal/
#48	animal model/
#49	exp rodent/
#50	(rat or rats or mouse or mice).ti.

#51	#43 or #44 or #45 or #46 or #47 or #48 or #49 or #50
#52	#35 not #51
#53	limit 52 to yr="2016 -Current"
