# Supplementary material for: Anti-hypertensive drugs deprescribing: an updated systematic review of clinical trials
Source: BMC Fam Pract. 2021 Oct 20;22:208. doi: 10.1186/s12875-021-01557-y (PMC8527765; doi:10.1186/s12875-021-01557-y)
Supplement: Supplementary file 3 — Additional file 3. .pdf: Excluded studies, with reasons for exclusion. [file 12875_2021_1557_MOESM3_ESM.docx]

Additional file 3. Excluded studies, with reasons for exclusion

| **Study** | **Reason for exclusion** |
| --- | --- |
| Alharbi et al., 2017 | Outcome not in line with PICOS framework |
| Buranakitjaroen et al., 2016 | Study design not in line with PICOS framework |
| Beeftink et al., 2017 | Study design not in line with PICOS framework |
| Woodhouse et al., 2017 | Population (i.e. patients with acute stroke) not in line with PICOS framework |
| Derk et al., 2018 | Full text not available |
| Gulla et al., 2018 | Outcome (i.e. effect of a medication review programme) not in line with PICOS framework |
| Kostev et al., 2019 | Outcome (i.e. dementia) not in line with PICOS framework |
| Authors not listed | Full text not available |
| Nambiar et al., 2019 | Study design not in line with PICOS framework |
| Schweber et al., 2019 | Outcome (i.e. evaluation of prevalence and predictors of early vs. later discontinuation) not in line with PICOS framework |
| Ecker-Schlipf et al., 2016 | Article not in English |
| Hirakawa et al., 2016 | Study design not in line with PICOS framework |
| Imprialos et al., 2016 | Outcome (i.e. decline in cognitive function) not in line with PICOS framework |
| Jongstra et al., 2016 | Study design not in line with PICOS framework |
| Moonen et al., 2016 | Study design not in line with PICOS framework |
| Babar et al., 2017 | Only protocol available |
| Jennings et al., 2017 | Study design not in line with PICOS framework |
| Whiting et al., 2017 | Outcome (i.e. risk of acute kidney injury) not in line with PICOS framework |
| de Jager et al., 2018 | Outcome (i.e. assessment of the causes of anti-hypertensive drugs non-adherence) not in line with PICOS framework |
| Legrand et al., 2019 | Only protocol available |
| Ku & Hsu et al., 2019 | Only protocol available |

**References**

Alharbi FF, Souverein PC, de Groot MC, Maitland-van der Zee AH, de Boer A, Klungel OH. Risk of acute myocardial infarction after discontinuation of antihypertensive agents: a case-control study. J Hum Hypertens. 2017 Aug;31(8):537-544.

Buranakitjaroen, P., Phoojaroenchanachai, M., Thongma, P., and Wimonsophonkitti, R. (2016). Effect of anti-hypertensive medication withdrawal in well-controlled treated hypertensive patients: Preliminary results. *J. Med. Assoc. Thail.*

Beeftink MMA, Van Der Sande NGC, Bots ML, Doevendans PA, Blankestijn PJ, Visseren FLJ, et al. Safety of Temporary Discontinuation of Antihypertensive Medication in Patients with Difficult-to-Control Hypertension. Hypertension. 2017.

Woodhouse LJ, Manning L, Potter JF, Berge E, Sprigg N, Wardlaw J, et al. Continuing or Temporarily Stopping Prestroke Antihypertensive Medication in Acute Stroke: An Individual Patient Data Meta-Analysis. Hypertension. 2017.

Gulla C, Flo E, Kjome RLS, Husebo BS. Deprescribing antihypertensive treatment in nursing home patients and the effect on blood pressure. J Geriatr Cardiol. 2018.

Kostev K, Bohlken J. Discontinuation of Antihypertensive Therapy and Dementia Incidence in the United Kingdom. J. Am. Med. Dir. Assoc. 2019.

Nambiar L, Silverman D, VanBuren P, LeWinter M, Meyer M. Beta-Blocker Cessation in Stable Outpatients with Heart Failure with a Preserved Ejection Fraction. J Card Fail. 2019.

Schweber AB, Ye S, Tajeu GS, Kronish IM. Prevalence and Predictors of Early Discontinuation From Antihypertensive Treatment Among New York City Medicaid Beneficiaries: the Case for Early Adherence Intervention and First-line Treatment With ACEi/ARBs [Internet]. Circulation. 2019. Available from: https://www.ahajournals.org/doi/10.1161/circ.140.suppl_1.11390

Hirakawa Y, Arima H, Webster R, Zoungas S, Li Q, Harrap S, et al. Risks associated with permanent discontinuation of blood pressure-lowering medications in patients with type 2 diabetes. J Hypertens. 2016.

Imprialos K, Boutari C, Doumas M. Discontinuation of antihypertensive treatment in elderly patients and cognitive function. JAMA Intern. Med. 2016.

Jongstra S, Harrison JK, Quinn TJ, Richard E. Antihypertensive withdrawal for the prevention of cognitive decline. Cochrane Database Syst. Rev. 2016.

Moonen JEF, Foster-Dingley JC, De Ruijter W, Van Der Grond J, De Craen AJM, Van Der Mast RC. Effect of discontinuation of antihypertensive medication on orthostatic hypotension in older persons with mild cognitive impairment: The DANTE Study Leiden. Age Ageing. 2016.

Babar ZUD, Gammie TM, Gnjidic D, Reeve E, Jordan V, Hilmer SN, et al. Withdrawal of antihypertensive drugs in older people. Cochrane Database Syst Rev. 2017.

Jennings GLR. Antihypertensive therapy: Strategic withdrawal or a mandatory life sentence? J. Hypertens. 2017.

Whiting P, Morden A, Tomlinson LA, Caskey F, Blakeman T, Tomson C, et al. What are the risks and benefits of temporarily discontinuing medications to prevent acute kidney injury? A systematic review and meta-Analysis. BMJ Open. 2017.

de Jager RL, van Maarseveen EM, Bots ML, Blankestijn PJ. Medication adherence in patients with apparent resistant hypertension: findings from the SYMPATHY trial. Br J Clin Pharmacol. 2018.

Legrand M, Futier E, Leone M, Deniau B, Mebazaa A, Plaud B, et al. Impact of renin-angiotensin system inhibitors continuation versus discontinuation on outcome after major surgery: Protocol of a multicenter randomized, controlled trial (STOP-or-NOT trial). Trials. 2019.

Ku E, Hsu R. ACEi ARB Withdrawal in CKD Patients [Internet]. NCT03957161. 2019. Available from: <https://clinicaltrials.gov/ct2/show/record/NCT03957161>
